# Prediction of Carcass Traits of Santa Inês Lambs Finished in Tropical Pastures through Biometric Measurements

**DOI:** 10.3390/ani11082329

**Published:** 2021-08-07

**Authors:** Antonio Leandro Chaves Gurgel, Gelson dos Santos Difante, João Virgínio Emerenciano Neto, Cynthia Gabriela Fernandes de Araújo, Marcone Geraldo Costa, Luís Carlos Vinhas Ítavo, Itania Maria Medeiros de Araujo, Carolina Marques Costa, Juliana Caroline Santos Santana, Camila Celeste Brandão Ferreira Ítavo, Patrick Bezerra Fernandes

**Affiliations:** 1 Faculty of Veterinary Medicine and Animal Science, Federal University of Mato Grosso do Sul, Campo Grande 79070-900, Brazil; gdifante@hotmail.com (G.d.S.D.); luis.itavo@ufms.br (L.C.V.Í.); itaniammaraujo@hotmail.com (I.M.M.d.A.); carolinaufgd@hotmail.com (C.M.C.); jukrol_@hotmail.com (J.C.S.S.); camila.itavo@gmail.com (C.C.B.F.Í.); zoo.patrick@hotmail.com (P.B.F.); 2 Academic Unit Specialized in Agricultural Sciences, Federal University of Rio Grande do Norte, Macaíba 59280-000, Brazil; joao_neto@zootecnista.com.br (J.V.E.N.); cynthiazootecnista@gmail.com (C.G.F.d.A.); marcogercosta@yahoo.com.br (M.G.C.)

**Keywords:** biometric measurements, carcass weight, forage, mathematical equations, primal cuts, Santa Inês, wool-less sheep

## Abstract

**Simple Summary:**

Biometric measurements have been used to estimate the composition and yield of sheep carcass cuts. However, for the most part, they are carried out with animals finished in feedlots and/or with released animals, which do not represent the reality of production systems located in tropical regions because tropical forage grasses are the food base of small and large ruminants and are responsible for most of the meat produced in the tropics. Therefore, the objective was to predict the weights of the primary carcass cuts of Santa Inês lambs finished in tropical pastures through biometric measurements. Adjustments of multiple linear equations and selection of variables to predict carcass characteristics were performed using the STEPWISE option and Mallow’s Cp. The biometric measurements obtained at the time of slaughter can be used as predictive variables of carcass of Santa Inês sheep finished in tropical pastures. The prediction equations are precise and accurate.

**Abstract:**

The aim of this study was to predict carcass traits of Santa Inês lambs finished in tropical pastures by using biometric measurements. Data originated from two experiments involving 56 lambs (32 in experiment I and 24 in experiment II). In both experiments, the sheep were finished in that were finished in pastures of *Panicum maximum* and *Brachiaria brizantha*, experiment I being conducted in the rainy season and experiment II in the dry season. The following biometric measurements were recorded before slaughter: body length (BL), withers height (WH), rump height (RH), thorax width (TW), rump width (RW), chest width (CW), heart girth (HG), thigh circumference (TC), rump circumference (RC) and leg length (LL), in addition to live weight at slaughter (SW). After slaughter, hot carcass weight (HCW), cold carcass weight (CCW) and the weights of primal cuts (shoulder, neck, loin, leg and rib) were recorded. In the equations generated to predict SW, HCW and CCW, R^2^ ranged from 0.58 to 0.91 and the measurements of WH, TC, CW, HG and RW were the most relevant. In the equations developed to predict the weight of primal cuts, in turn, R^2^ ranged from 0.26 to 0.99. In these models, SW, BL, CW, TC, LL and HG explained most of the variation in the weight of primal cuts. Biometric measurements can be used to accurately and precisely predict HCW, CCW and the weight of primal cuts from the carcass of Santa Inês sheep finished in tropical pastures, since the equations presented R^2^ and correlation coefficient and agreement above 0.8.

## 1. Introduction

Various methods have been used to determine carcass traits of ruminant animals [1,2,3,4]. In addition to being time-consuming and costly due to the high number of samples and laboratory analyses required, these techniques promote waste, since half of the carcass is discarded after evaluations [5]. In this scenario, the use of biometric measurements can be a non-invasive and viable alternative to estimate carcass traits in sheep, as it has little to no additional cost to producers [6,7,8].

Biometric measurements have been used to estimate the composition and yield of cuts from sheep carcasses [9,10,11]. Recently, Gomes et al. [12] reported that morphometric measurements can be used in conjunction with animal weight to increase the accuracy of predictive equations for the characteristics of Santa Inês sheep carcasses. Likewise, Costa et al. [7] and Bautista-Díaz et al. [6] showed that biometric measurements can be used to predict the carcass characteristics of feedlot-finished lambs. However, in their vast majority, these measurements are performed on animals finished in feedlots and/or on wool sheep [13], which do not represent the reality of production systems in tropical regions, since tropical forage grasses are the food base of small and large ruminants and are responsible for most of the meat produced in the tropics [14].

The Santa Inês breed originated in the northeast region of Brazil from accidental crosses between the Bergamácia, Morada Nova, and Somalis breeds, and animals without a defined breed standard [15]. Santa Inês are medium- to large-framed sheep with high maternal ability. Despite producing more milk than other native wool-less breeds, it is useful for meat and leather production [15]. Because it is adapted to environments with high temperatures and prolonged periods of drought, it is recommended for more extensive production systems.

Given the low adoption of biometric measurements to estimate the carcass traits of wool-less sheep finished in tropical pastures, the present study investigated the hypothesis that these measurements can be used as predictors of carcass weight and primal cuts from the carcass of Santa Inês sheep finished in tropical pastures. Thus, the aim was to predict the carcass traits of Santa Inês lambs finished in tropical pastures by using biometric measurements.

## 2. Materials and Methods

The data used in the predictions originated from two experiments conducted at the Federal University of Rio Grande do Norte, located in Macaíba, RN—Brazil (5°53′34″ S 35°21′50″ W, 50 m above sea level). In experiment I, from April to September 2011 [16,17], 32 castrated male Santa Inês sheep at 90 days of age, with an average live weight of 23.8 ± 1.6 kg, were randomly divided into four groups of eight animals that were allocated to four pastures of tropical grasses (two *Panicum maximum* cultivars: Aruana and Massai; and two *Brachiaria brizantha* cultivars: Marandu and Piatã). The pastures were managed in an intermittent grazing system with pre-grazing and post-grazing target heights of 50 cm and 25 cm, respectively.

In experiment II, from October 2011 to January 2012 [18], 24 castrated male Santa Inês sheep at 90 days of age, with an average live weight of 23.8 ± 2.0 kg, were randomly divided into four groups of six animals that were allocated to the same tropical grass pastures used in experiment I. The pastures were managed in an intermittent grazing system with seven days of occupation and 35 days of rest. Because the experiment was conducted in the dry season of the year, the animals received concentrate supplementation (39.1% ground maize, 30.0% cottonseed cake, 25.1% soybean meal, 3.0% mineral supplement and 2.8% livestock urea) at a rate of 1.38% of their live weight. In both experiments, the animals were kept on pasture during the daytime (from 07:00 to 16:00) and housed in a shed with collective stalls at night, where they had ad libitum access to water and mineral salt.

The following biometric measurements were recorded for each animal 24 h before slaughter, following Cézar and Sousa [19]: body length (BL), withers height (WH), rump height (RH), thorax width (TW), rump width (RW), chest width (CW), heart girth (HG), thigh circumference (TC), rump circumference (RC), and leg length (LL). A tape measure and a measuring stick were used to perform the measurements.

The slaughter criterion in both experiments was live weight. Upon reaching approximately 32 kg of live weight, the sheep were fasted for 16 h to record live weight at slaughter (SW). At the time of slaughter, the animals were stunned in the atlanto-occipital region, followed by bleeding through carotid and jugular sections. After skinning and evisceration, the head (section at the atlanto-occipital joint) and extremities (section at the metacarpal and metatarsal joints) were removed.

The carcasses were then weighed to determine the hot carcass weight (HCW). Subsequently, they were transferred to a cold room at 5 °C, where they were kept for 24 h hung by the tendons on appropriate hooks, at a distance of 17 cm between the tarsometatarsal joints. After this period, the carcasses were weighed again (cold carcass weight, CCW).

Next, the carcasses were cut in half and the left half was weighed and subdivided into five anatomical regions that were weighed individually, in an adaptation of the methodology proposed by Osório et al. [20]. The following primal cuts were thus obtained: shoulder, neck, loin, leg and rib.

A descriptive statistical analysis was performed using the PROC SUMMARY procedure of SAS software (SAS University Edition, Sas Institute Inc., Cary, CA, USA). Pearson’s correlation coefficients between variables were estimated using the PROC CORR procedure of SAS (SAS University Edition, Sas Institute Inc., Cary, CA, USA). Model adjustments and selection of variables were carried out using the PROC REG procedure of SAS (SAS University Edition, Sas Institute Inc., Cary, CA, USA). The STEPWISE option and Mallow’s Cp were used to select the variables to be included in the equations. The outliers were tested by evaluating the studentized residuals in relation to the values predicted by the equations. Residuals that were outside the range of −2.5 to 2.5 were excluded. The goodness of fit of the developed equations was assessed based on the coefficient of determination (R^2^) and the root mean squared error (RMSE). For each predicted carcass characteristic more than one equation was generated, totaling 26 equations. Thus, the equation that obtained the lowest RMSE and highest R^2^ was recommended.

The data estimated by the equations that obtained the best fits were compared with the real values, using the following regression model:

Y = β0 + β1 × X + εi

where Y is the observed value; β0 and β1 represent the intercept and the slope of the regression equation, respectively; X is the value predicted by the equations and εi is the error associated with that observed in the response variable. The criteria for evaluating the adequacy of the equations were as follows: coefficient of determination (R^2^); F test for the identity of the parameters (β0 = 0 and β1 = 1) of the regression of predicted on observed data; concordance correlation coefficient (CCC); root mean square error of prediction (RMSEP); and decomposition of the mean square error of prediction (MSEP) into mean error, systematic bias and random error [21], using Model Evaluation System software version 3.2.2 (http://nutritionmodels.tamu.edu/mes.htm, College Station, TX, USA). A significance level of 5% was adopted for all statistical analyses.

## 3. Results and Discussion

Slaughter weight had a coefficient of variation (CV) of 8.17%. Accordingly, HCW and CCW ranged from 9.92 to 16.14 kg, both with a CV around 10.6% (Table 1). Of all primal cuts extracted from the carcass, the neck and loin showed the highest CV (18.31 and 17.11%, respectively). Except for RW, which exhibited a high CV (24.96%), the other biometric measurements showed variations of low to moderate magnitude (5.62–12.25%). This low variability in the dataset may be associated with the behavior shown by SW. Although the database corresponded to two experiments with different experimental conditions, the animals were slaughtered at the average weights of 32.72 ± 2.82 and 33.69 ± 0.49 kg (experiments I and II, respectively) [16,17,18].

Slaughter weight was positively correlated with BL, WH, CW, and TC (Table 2). Three equations were obtained to predict SW, with R^2^ ranging from 0.58 to 0.76 (Table 3). These models included WH, TC, and HG (*p* < 0.05). The quadratic value of these variables was tested and revealed that, when squared, HG provided an increase in R^2^ and a reduction in RMSE (Equation (3)). Because the WG, TC, and HG variables showed high correction with weight, these variables were included in the equation that showed greater predictive capacity (Equation (3)). Studies with ruminants of different species, breeds and sexes and wide variations in weight and age also showed that heart girth is an efficient measurement to estimate live weight [7,22,23].

Hot and cold carcass weight correlated with BL, RW, CW, and TC. In the equations generated to predict these two variables, R^2^ ranged from 0.68 to 0.91 (HCW) and 0.67 to 0.95 (CCW). The variables of SW, CW, RW, TC, BL, and LL were included in these models (Table 3). Slaughter weight was responsible for a high percentage of the variation in HCW and CCW (Table 3). Several authors claim that SW is the main responsible for variations in other carcass measurements [3,7,24,25]. However, the inclusion of biometric measurements in the prediction equations increased R^2^ and reduced RMSE (Table 3). Therefore, the best models were obtained when biometric measurements were included.

These results agree with those published by other researchers [26,27], who showed that the measurements of BL, CW, and RW can be used to predict the HCW of wool lambs. Costa et al. [8] found that BL and CW are important variables for predicting HCW and CCW in Morada Nova sheep. Shehata [10] reported that BL predicts 79% of the variation in HCW and CCW. Pinheiro and Jorge [28] recommended using CW and RW to more accurately estimate the weight of Santa Inês sheep carcasses.

Shoulder weight was positively correlated with CW and TC (Table 2). Thus, four prediction equations were obtained, in which R^2^ ranged from 0.56 to 0.99 (Table 4). The most relevant biometric measurements in the formulation of these equations were BL, WH, CW, TC and RC, in addition to SW. The quadratic value of these variables did not improve the equation. The intercept became insignificant (*p* > 0.05) during the variable-selection process and was thus excluded from the final equation. These results are similar to those described by Abdel-Moneim [10] and Shehata [11], who stated that BL, CW, WH, and SW are significant variables and should be included in models for predicting shoulder weight in wool sheep.

None of the biometric measurements correlated with neck weight (*p* > 0.05). This can be explained, in part, by the fact that no biometric measurement is taken from this anatomical region [13]. For this cut, only two equations were generated, in which R^2^ ranged from 0.23 to 0.99 and the variables of SW, BL and TC were included (Table 4). No studies were found using biometric measurements to predict neck weight in sheep; nonetheless, the variables included in the equation are consistent with the observed correlation coefficients (Table 2).

The equations to predict loin weight showed R^2^ ranging from 0.55 to 0.81. Slaughter weight, BL, TW, CW, and HG were included in these models (Table 4). Because the loin is a meat cut extracted from the *Longissimus dorsi* muscle, which runs along the animal’s back, it is correlated with BL and with all measurements associated with muscle deposition (Table 4). Thus, when the biometric measurements were entered in the models, R^2^ increased by 0.26. Heart girth and BL can explain 45 and 50%, respectively, of the variation in loin weight in sheep [11].

Three equations were generated to predict leg weight. In this case, SW associated with the CW and TC were selected (*p* < 0.05) as predictive variables. “Leg”, the most important of all primal cuts of the sheep carcass [29], consists mainly of the muscles that surround the femur [30]. This is the region where TC is measured, which explains its direct relationship with leg weight (Table 1).

The equations for predicting rib weight were fitted using the measurements of HG and CW. This is in line with the anatomical position of the ribs in sheep carcasses, since HG is a measurement taken around the thoracic cavity and CW consists of the distance between the acromions [13]. Nigm et al. [9] also concluded that HG was the best measurement to predict rib weight in Merino sheep. Additionally, in buffalo calves, Rashad et al. [31] found that HG was highly correlated with all carcass traits.

In the evaluation of the selected equations (Table 5), all exhibited an R^2^ greater than 0.85, except Equation (16). In addition, with the exception of Equation (16), all were in agreement with the observed data (CCC > 0.85). Based on the RMSEP, the models show good ability to predict the exact weight of the variables. The carcass trait estimates were equal (*p* > 0.05) to the observed data (β0 = 0 and β1 = 1). Finally, the decomposition of the mean squared error of prediction showed that over 98% of error in the equations has a random origin.

Therefore, the obtained results confirm the hypothesis that biometric measurements can be used as predictive variables for HCW, CCW and primal cuts of the carcass of Santa Inês sheep finished in tropical pastures. 

## 4. Conclusions

The biometric measurements obtained at the time of slaughter can be used as predictive variables of HCW, CCW, and primal cuts of the carcass of Santa Inês sheep finished in tropical pastures. The prediction equations are precise and accurate.

Thus, these equations can be used by producer researchers, technicians and the meat industry to obtain information on the carcass characteristics of wool-less lambs before the animals are slaughtered. However, it is necessary that more research be carried out with the objective of increasing the database so that the equations can be applied in the most diverse scenarios. It is important that biometric measurements be taken from the neck region so that better models are fitted to predict this cut.

## Figures and Tables

**Table 1:** Summarized descriptive statistics of the parameters obtained in vivo and on the carcass of wool-less sheep finished in tropical pastures.

Variable	Description	*n*	Mean ± SD	Minimum	Maximum	CV (%)
SW (kg)	Live weight at slaughter	56	32.13 ± 1.89	28.00	36.60	8.17
HCW (kg)	Hot carcass weight	56	12.86 ± 1.37	10.50	16.14	10.65
CCW (kg)	Cold carcass weight	56	12.28 ± 1.30	9.92	15.28	10.59
Shoulder (kg)	Shoulder weight	56	1.12 ± 0.13	0.90	1.48	11.61
Neck (kg)	Neck weight	56	0.71 ± 0.13	0.43	1.16	18.31
Loin (kg)	Loin weight	56	0.76 ± 0.13	0.54	1.12	17.11
Leg (kg)	Leg weight	56	1.99 ± 0.22	1.62	2.48	11.05
Rib (kg)	Rib weight	56	1.53 ± 0.22	1.18	2.10	14.38
BL (cm)	Body length	56	60.58 ± 5.21	52.00	69.00	8.60
WH (cm)	Withers height	56	66.65 ± 4.38	56.00	74.00	6.57
RH (cm)	Rump height	56	70.36 ± 5.11	61.00	79.00	7.26
TW (cm)	Thorax width	56	27.27 ± 2.77	21.00	32.00	10.16
RW (cm)	Rump width	56	19.59 ± 4.89	14.00	30.00	24.96
CW (cm)	Chest width	56	17.52 ± 1.15	16.00	20.00	6.56
HG (cm)	Heart girth	56	87.26 ± 8.47	71.00	97.50	9.70
TC (cm)	Thigh circumference	56	37.21 ± 4.56	30.00	51.00	12.25
RC (cm)	Rump circumference	56	84.70 ± 4.88	67.00	94.00	5.76
LL (cm)	Leg length	56	33.79 ± 1.90	28.00	37.00	5.62

*n* = number of observations; SD = standard deviation; CV: coefficient of variation. SW: Live weight at slaughter; HCW: Hot carcass weight; CCW: Cold carcass weight; BL: Body length; WH: Withers height; RH: Rump height; TW: Thorax width; RW: Rump width; CW: Chest width; HG: Heart girth; TC: Thigh circumference; RC: Rump circumference; LL: Leg length.

**Table 2:** Pearson’s correlation coefficients between biometric measurements and carcass traits of sheep finished in tropical pastures.

	SW	HCW	CCW	Shoulder	Neck	Loin	Leg	Rib	BL	WH	RH	TW	RW	CW	HG	TC	RC	LL
SW	1	0.82 *	0.82 *	0.62 *	0.05	0.74 *	0.75 *	0.67 *	0.42 *	0.55 *	0.4	0.23	0.23	0.48 *	0.38	0.49 *	0.36	0.31
HCW		1	0.99 *	0.85 *	0.12	0.81 *	0.90 *	0.75 *	0.43 *	0.39	0.32	0.12	0.39	0.73 *	0.32	0.45 *	0.2	0.02
CCW			1	0.86 *	0.17	0.81 *	0.91 *	0.73 *	0.43 *	0.38	0.28	0.11	0.42 *	0.74 *	0.29	0.46 *	0.22	−0.03
Shoulder				1	0.19	0.59 *	0.82 *	0.42 *	0.17	0.26	0.24	0.05	0.19	0.75 *	0.1	0.45 *	0.26	−0.04
Neck					1	−0.04	0.24	−0.07	0.25	−0.02	0.24	0.06	0.16	0.2	−0.16	0.1	−0.03	−0.36
Loin						1	0.63 *	0.69 *	0.53 *	0.40 *	0.38	0.04	0.47 *	0.50 *	0.45 *	0.3	0.15	0.2
Leg							1	0.64 *	0.43 *	0.35 *	0.24	0.24	0.36	0.69 *	0.2	0.41 *	0.24	−0.01
Rib								1	0.48 *	0.42 *	0.40 *	0.34	0.36	0.37	0.67 *	0.11	0.11	0.18
BL									1	0.34	0.34	0.37	0.4	0.17	0.35	−0.07	0.11	0.19
WH										1	0.71 *	0.37	0.05	0.01	0.36	−0.06	0.58 *	0.38
RH											1	0.13	0.05	0.02	0.26	−0.13	0.38	0.60 *
TW												1	0.13	−0.11	0.61 *	−0.21	0.28	0.37
RW													1	0.27	0.13	0.29	0.02	−0.05
CW														1	−0.17	0.76 *	−0.1	−0.36
HG															1	−0.27	0.13	0.55 *
TC																1	0.01	−0.28
RC																	1	0.23
LL																		1

Correlations followed by no superscript indicate no significance; * *p* < 0.05. SW: Live weight at slaughter; HCW: Hot carcass weight; CCW: Cold carcass weight; BL: Body length; WH: Withers height; RH: Rump height; TW: Thorax width; RW: Rump width; CW: Chest width; HG: Heart girth; TC: Thigh circumference; RC: Rump circumference; LL: Leg length.

**Table 3:** Regression equations for predicting carcass traits of sheep finished in tropical pastures through biometric measurements. Values in parentheses are the PEs of the parameter estimates.

Equation No.	Equation	RMSE	R^2^	*p*-Value
SW				
(1)	SW (kg) = −17.51 (±9.30 *) + 0.49 (±0.12 *) × WH + 0.54 (±0.14 *) × TC	1.49	0.58	<0.0001
(2)	SW (kg) = −21.72 (±8.15 *) + 0.38 (±0.11 *) × WH + 0.10 (±0.03 *) × HG + 0.64 (±0.13 *) × TC	1.28	0.71	<0.0001
(3)	SW (kg) = −101.62 (±27.36 *) + 0.32 (±0.10) × WH + 0.60 (±0.12 *) × TC + 2.14 (±0.94 *) × HG − 0.01 (±0.005 *) × HG^2^	1.17	0.76	<0.0001
HCW				
(4)	HCW (kg) = −3.09 (±2.48 *) + 0.51 (±0.08 *) × SW	0.80	0.68	<0.0001
(5)	HCW (kg) = −7.22 (±2.19 *) + 0.38 (±0.07 *) × SW + 0.48 (±0.12 *) × CW	0.62	0.81	<0.0001
(6)	HCW (kg) = −7.52 (±2.05 *) + 0.41 (±0.05 *) × SW + 0.28 (±0.11 *) × RW + 0.77 (±0.12 *) × CW − 0.28 (±0.17 *) × TC	0.45	0.91	<0.0001
(7)	HCW (kg) = −3.58 (±1.48 *) + 0.41 (±0.05 *) × SW + 0.02 (±0.003 *) × CW^2^ − 0.004 (±0.0009 *) × TC^2^ + 0.009 (±0.004 *) × RW^2^	0.45	0.91	<0.0001
CCW				
(8)	CCW (kg) = −2.36 (±2.31 *) + 0.47 (±0.07 *) × SW	0.75	0.67	<0.0001
(9)	CCW (kg) = −4.04 (±1.72 *) + 0.38 (±0.05 *) × SW + 0.76 (±0.12 *) × CW − 0.24 (±0.07 *) × TC	0.45	0.89	<0.0001
(10)	CCW (kg) = −1.23 (±2.84 *) + 0.48 (±0.05 *) × SW − 0.05 (±0.03 *) × BL + 0.37 (±0.09 *) × RW + 0.68 (±0.01 *) × CW − 0.33 (±0.06 *) × TC − 0.12 (±0.05 *) × LL	0.32	0.95	<0.0001

*: *p* < 0.05. SW: live weight at slaughter (kg); HCW: hot carcass weight (kg); CCW: cold carcass weight (kg); WH: withers height (cm); TC: thigh circumference (cm); HG: heart girth (cm); CW: chest width (cm); RW: rump width (cm); BL: body length (cm); LL: leg length (cm); RMSE: root mean square error; R^2^: coefficient of determination.

**Table 4:** Regression equations for predicting the weights of primal cuts from the carcass of wool-less sheep finished in tropical pastures through biometric measurements. Values in parentheses are the PEs of the parameter estimates.

Equation No.	Equation	RMSE	R^2^	*p*-Value
Shoulder				
(11)	Shoulder (kg) = −0.49 (±0.31 *) + 0.09 (±0.02 *) × CW	0.10	0.56	<0.0001
(12)	Shoulder (kg) = −1.14 (±0.42 ^ns^) + 0.13 (±0.02 *) × CW − 0.03 (±0.01 *) × TC + 0.01 (±0.003 *) × RC	0.09	0.72	<0.0001
(13)	Shoulder (kg) = 0.04 (±0.01 *) × SW − 0.01 (±0.005 *) × BL + 0.12 (±0.02 *) × WH + 0.13 (±0.02 *) × CW − 0.05 (±0.01 *) × TC + 0.009 (±0.004) × RC	0.08	0.99	<0.0001
(14)	Shoulder (kg) = 0.12 (±0.02 *) × CW − 0.03 (±0.01 *) × TC + 0.0006 (±0.0001 *) × SW^2^ − 0.0001 (±0.00005 *) × BL^2^	0.08	0.99	<0.0001
Neck				
(15)	Neck (kg) = 0.54 (±0.33 ^ns^) + 0.02 (±0.005 *) × BL − 0.02 (±0.008 *) × LL	0.07	0.23	0.0583
(16)	Neck (kg) = 0.014 (±0.0008 *) × SW + 0.02 (±0.004 *) × BL − 0.018 (±0.007 *) × LL	0.07	0.99	<0.0001
Loin				
(17)	Loin (kg) = −0.62 (±0.26 *) + 0.04 (±0.009 *) × SW	0.09	0.55	<0.0001
(18)	Loin (kg) = −1.02 (±0.36 *) + 0.04 (±0.009 *) × SW + 0.01 (±0.006 *) × BL	0.09	0.60	<0.0001
(19)	Loin (kg) = −0.88 (±0.34 *) + 0.02 (±0.009 *) × SW + 0.01 (±0.005 *) × BL − 0.04 (±0.01 *) × TW + 0.04 (±0.01 *) × CW + 0.008 (±0.002 *) × HG	0.06	0.81	<0.0001
Leg				
(20)	Leg (kg) = −0.77 (±0.41^ns^) + 0.05 (±0.01 *) × SW + 0.07 (±0.02 *) × CW	0.11	0.69	<0.0001
(21)	Leg (kg) = 0.05 (±0.01 *) × SW + 0.12 (±0.02 *) × CW − 0.05 (±0.01 *) × TC	0.10	0.99	<0.0001
(22)	Leg (kg) = 0.05 (±0.006 *) × SW + 0.003 (±0.0007 *) × CW^2^ − 0.0005 (±0.0002 *) × TC^2^	0.10	0.99	<0.0001
Rib				
(23)	Rib (kg) = −1.17 (±0.45 *) + 0.08 (±0.02 *) × CW + 0.02 (±0.003 *) × HG	0.12	0.70	<0.0001
(24)	Rib (kg) = −2.15 (±0.68 *) + 0.02 (±0.009 *) × RH + 0.08 (±0.02 *) × CW + 0.02 (±0.003 *) × HG	0.11	0.74	<0.0001
(25)	Rib (kg) = 0.41 (±0.36 ^ns^) + 0.08 (±0.003 *) × HG + 0.003 (±0.0007 *) × CW^2^	0.14	0.58	<0.0001
(26)	Rib (kg) = 0.13 (±0.001 *) × HG + 0.002 (±0.0004 *) × CW^2^	0.10	0.99	<0.0001

*: *p* < 0.05; ns: Not significant; CW: chest width (cm); TC: thigh circumference (cm); RC: rump circumference (cm); SW: live weight at slaughter (kg); BL: body length (cm); WH: withers height (cm); LL: leg length (cm); TW: thorax width (cm); HG: heart girth (cm); RH: rump height (cm); RMSE: root mean square error; R^2^: coefficient of determination.

**Table 5:** Mean and descriptive statistics of the fit of equations for predicting carcass traits of sheep in finished tropical pastures through biometric measurements.

Variable	Equation (3) SW	Equation (6) HCW	Equation (9) CCW	Equation (13) Shoulder	Equation (16) Neck	Equation (19) Loin	Equation (23) Leg	Equation (26) Rib
Mean	32.73	13.68	13.07	1.15	0.73	0.84	2.07	1.65
Standard deviation	1.92	1.31	1.24	0.14	0.07	0.12	0.17	0.13
Minimum	28.57	11.46	10.81	0.94	0.64	0.58	1.75	1.43
Maximum	35.97	16.35	15.34	1.52	0.90	1.07	2.42	1.90
CCC	0.87	0.95	0.98	0.89	0.66	0.90	0.86	0.79
RMSEP	3.12	2.92	2.08	5.75	8.42	6.77	4.65	6.11
R^2^	0.87	0.95	0.98	0.90	0.68	0.90	0.87	0.92
Regression analysis								
Intercept (β0)	−0.00008	−0.004	0.00002	−0.10	0.09	0.00006	−0.04	−0.30
Slope (β1)	1.00	1.00	1.00	1.01	0.88	0.99	1.02	1.18
*p*-value (β0 = 0 and β1 = 1)	1.00	0.99	1.00	0.99	0.84	1.00	0.98	0.56
Decomposition of MSEP (%)								
Mean bias	0.00	0.00	0.00	0.00	0.01	0.00	0.00	0.03
Systematic bias	0.00	0.00	0.00	0.04	1.64	0.00	0.10	5.34
Random error	100.00	99.99	100.00	99.96	98.35	100.00	99.90	94.62

SW: live weight at slaughter (kg); HCW: hot carcass weight (kg); CCW: cold carcass weight (kg); CCC = concordance correlation coefficient; RMSEP = root mean square error of prediction; R^2^: coefficient of determination; MSEP: mean square error of prediction.

## Data Availability

Not applicable.
